# Multiple Electric Energy Consumption Forecasting Using a Cluster-Based Strategy for Transfer Learning in Smart Building

**DOI:** 10.3390/s20092668

**Published:** 2020-05-07

**Authors:** Tuong Le, Minh Thanh Vo, Tung Kieu, Eenjun Hwang, Seungmin Rho, Sung Wook Baik

**Affiliations:** 1 Informetrics Research Group, Ton Duc Thang University, Ho Chi Minh City 700000, Vietnam; lecungtuong@tdtu.edu.vn; 2 Faculty of Information Technology, Ton Duc Thang University, Ho Chi Minh City 700000, Vietnam; 3 Institute of Research and Development, Duy Tan University, Da Nang 550000, Vietnam; vmthanhit@gmail.com; 4 University of Science, Vietnam National University, Ho Chi Minh City 700000, Vietnam; kvttung@gmail.com; 5 School of Electrical Engineering, Korea University, Seoul 02841, Korea; ehwang04@korea.ac.kr; 6 Department of Software, Sejong University, Seoul 05006, Korea; smrho@sejong.edu

**Keywords:** multiple electric energy consumption forecasting, long short-term memory networks, transfer learning, the cluster-based strategy for transfer learning, intelligent energy management system

## Abstract

Electric energy consumption forecasting is an interesting, challenging, and important issue in energy management and equipment efficiency improvement. Existing approaches are predictive models that have the ability to predict for a specific profile, i.e., a time series of a whole building or an individual household in a smart building. In practice, there are many profiles in each smart building, which leads to time-consuming and expensive system resources. Therefore, this study develops a robust framework for the Multiple Electric Energy Consumption forecasting (MEC) of a smart building using Transfer Learning and Long Short-Term Memory (TLL), the so-called MEC-TLL framework. In this framework, we first employ a k-means clustering algorithm to cluster the daily load demand of many profiles in the training set. In this phase, we also perform *Silhouette* analysis to specify the optimal number of clusters for the experimental datasets. Next, this study develops the MEC training algorithm, which utilizes a cluster-based strategy for transfer learning the Long Short-Term Memory models to reduce the computational time. Finally, extensive experiments are conducted to compare the computational time and different performance metrics for multiple electric energy consumption forecasting on two smart buildings in South Korea. The experimental results indicate that our proposed approach is capable of economical overheads while achieving superior performances. Therefore, the proposed approach can be applied effectively for intelligent energy management in smart buildings.

## 1. Introduction

Nowadays, many applications of artificial intelligence have been developed in various areas, such as business intelligence [1,2,3,4], intelligent systems in construction [5,6], medical and health care [7,8], trash classification [9], facial analysis [10,11,12], intelligent energy management system [13,14], and energy consumption forecasting [15,16]. Recently, energy consumption forecasting has been attracting massive research interest due to the importance of the sustainable environment as well as the benefits brought to consumers and suppliers. Electrical energy consumption forecasting can be considered as the most crucial task in the intelligent energy management domain. Electrical energy consumption forecasting can be employed as users’ energy demands. Therefore, electrical energy consumption forecasting was usually implemented as an incorporated module in energy management systems [17]. Then, based on the forecasting values, suppliers may change the production of electric energy generators. Likewise, suppliers can detect abnormal usage patterns, which deviate totally from the predicted values [18]. These abnormal patterns probably indicate energy leakages.

There are two main approaches that use Machine Learning to solve the electrical energy consumption forecasting problem in smart buildings, including occupant-centric and energy/devices-centric [17]. The first approach considers occupant aspects, including occupancy estimation and identification, human activity recognition, and preference estimation. In contrast, the second approach considers device and energy aspects, including energy profiling, demand estimation, devices profiling, and inference on sensors. The first approach employs both users’ information in buildings (e.g., the number of people, gender, and usage type) and sensors’ information (e.g., humidity, temperature, and brightness) to estimate the energy consumption [19]. Human behaviors, such as meeting schedules [20], sleep patterns [21], movement patterns [22], and daily activities [23], are also harvested to improve the quality of the estimation. In addition, user preferences such as a comfortable temperature [24] and friendly brightness [25] are employed to enhance learning models. The second approach directly considers the energy consumption of devices, such as appliances [26], devices’ functions [27], interview-based load profiles [28], and individual apartment/building profiles [29], to build an estimator. It is worth noting that a large number of machine learning algorithms are applied to both aforementioned approaches, such as *k* Nearest Neighbors (kNN), Support Vector Machines (SVMs), and Neural Networks (NNs). Among all algorithms, ANNs dominate the number of studies due to their impressive performance. Recently, inspired by biological processes, a totally new approach, so-called energy homeostasis, has been proposed to build a complete energy management system where consumption prediction is an incorporated module [30,31]. In this paper, we propose an energy-centric-based method for electric energy consumption using an NN-based method.

Currently, many studies have utilized NNs, especially a specialized recurrent unit, namely Long Short-Term Memory networks (LSTMs), to forecast electric energy consumption. However, they only focus on forecasting for a specific profile, i.e., the electric energy consumption of an individual apartment in smart buildings. In practice, a smart building has many apartments; hence, there are a large number of profiles. The existing models are not effective in terms of computational time for forecasting multiple profiles because it repeats the training process with different profiles. Therefore, this study develops an effective framework for multiple electric energy consumption forecasting in smart buildings by using a cluster-based strategy for transfer learning between LSTM models to reduce the computational time. To the best of our knowledge, this is the first study that employs a cluster-based strategy and transfer learning to improve performances and reduce computational overheads of LSTMs for electrical energy consumption forecasting. The main contributions of our research are as follows: (1) This study develops a time series clustering module using a k-means clustering algorithm to divide the training dataset into disjoint clusters. (2) A Multiple Electric Energy Consumption forecasting (MEC) training algorithm is then introduced. (3) Next, an effective framework for multiple electric energy consumption forecasting is developed by using transfer learning and cluster-based strategies for training LSTM models to reduce the computational time. (4) Finally, experiments are conducted on the energy consumption datasets of two smart buildings located in South Korea. The experimental results indicate that the MEC-TLL framework outperforms two baseline approaches, including a traditional machine learning approach and an approach that employs transfer learning without the cluster-based strategy in terms of the computational time, while retaining a predictive performance based on Mean Absolute Error (*MAE*), Root Mean Square Error (*RMSE*), and Mean Absolute Percentage Error (*MAPE*) metrics.

The remaining of this article is structured as follows. Section 2 introduces related studies on electric energy consumption prediction and time series prediction. Section 3 summarizes the basic concepts, including *k*-means clustering algorithm, LSTM networks for time series analysis, and transfer learning. The proposed framework named MEC-TLL for multiple electric energy consumption forecasting, is introduced in Section 4. Next, Section 5 shows the first experiment to select the optimal number of clusters for each dataset. Besides, Section 5 also provides the experimental results of the experimental approaches for multiple electric energy consumption forecasting on two energy consumption datasets collected from two smart buildings in South Korea. The conclusions and several future directions are presented in Section 6.

## 2. Related Works

### 2.1. Electric Energy Consumption Prediction

Hebrail and Berard [32] release an individual household electric power consumption (*IEC*) dataset available on the *UCI Machine Learning Repository*. This dataset is collected from an individual house located in France. The dataset is utilized in many research studies. Kim and Cho [15,16] develop two efficient models to predict the electric energy consumption for the *IEC* dataset; The former [15] proposes a machine learning approach that can be explained by not only predicting future electric energy consumption but also identifying the current demand patterns. The latter [16] proposes an effective model, namely CNN-LSTM, that combines Convolutional Neural Networks (CNNs) with LSTMs to extract spatial and temporal features, which in turn stably predict energy consumption. Le et al. [33] develop the EECP-CBL model, which is a combination of CNNs and Bi-directional Long Short-Term Memory networks (Bi-LSTMs). The experimental results in [33] indicated that EECP-CBL is better than state-of-the-art models in terms of accuracy and computational time on the *IEC* dataset with various timespan settings. In addition, there are several other interesting studies [34,35,36,37,38] for building energy consumption on other datasets. Tian et al. [34] utilize the parallel learning theory to develop a parallel prediction strategy for building energy consumption forecasting. Specifically, they utilize Generative Adversarial Networks (GANs), which comprise two adversarial sub-models, including a generator and a discriminator. The experimental results in this study indicate that their proposed approach outperforms state-of-the-art methods on two real-world datasets, including a retail building in Fremont, CA and a new-built commercial office building located in Beijing. Yan et al. [35] introduce a hybrid deep learning model, which combines an ensemble model of LSTMs with a stationary wavelet transform technique to improve the predictive performance on five different family houses’ energy consumption datasets in London. Wang et al. [36] propose a novel integration model for building energy prediction on two educational buildings in Tianjin, China. Park et al. [37] propose a two-stage short-term demand prediction (STDP) model that combines popular STDP models by using a deep neural network, thus further expanding the domain of applicability. To demonstrate the proposed model performance, the authors compare several machine learning methods with the proposed approach for one-day-ahead forecasting on a factory electric energy consumption dataset. Liu et al. [38] utilize three famous deep reinforcement learning techniques, including Advantage Actor-Critic, Deep Deterministic Policy Gradient (DDPG), and Recurrent Deterministic Policy Gradient (RDPG) for the problem of building energy consumption forecasting on an office building located in Henan, China. The experiment study shows that DDPG and RDPG are the best approaches in terms of predictive performance. A critical disadvantage of DDPG and RDPG is that they have a large computational time.

### 2.2. Time Series Prediction

The problem of time series prediction is considered as the most important problem in machine learning, with a large number of practical applications such as stock price trend prediction [39], housing price prediction [40], sensor data analysis [41], and water price prediction [42]. LSTMs are the most popular specialized model of recurrent neural networks (RNNs) for the time series forecasting problem. LSTMs are better than traditional RNNs because LSTMs are capable of learning long-term dependencies. Unlike traditional RNN models, which usually face the problem of vanishing gradients on long sequential data, LSTM overcomes the vanishing gradient problem by introducing three gates, including the input gate, output gate, and forget gate, in each cell. These gates have the ability to capture the temporal changes for extremely long sequential data. Because of its advantages, it has been utilized widely in various applications such as text [43], videos [44], time series analysis [39,40], traffic forecast [45], speech recognition [46], and time series anomaly detection [47]. Lin et al. [43] introduce an application of LSTMs on the task of mention extraction, where LSTMs extract and classify overlapped and nested structure mentions. Dai et al. [44] utilize LSTMs to propose a two-stream attention-based LSTM approach for the problem of action recognition in videos. In addition, Ta et al. [39] utilize LSTMs to predict stock movement based on historical data. Recently, Liu et al. [40] developed an LSTM approach that incorporated a modified genetic algorithm with multi-level probability crossover to select appropriate features and the optimal hyper-parameters to predict the housing price of a city by using historical data. This model was verified on a housing price dataset in Shenzhen, China. The results confirmed that their approach has a good performance in modeling housing prices and obviously outperforms state-of-the-art algorithms. Zhao et al. [45] apply LSTMs to the problem of traffic forecast to achieve better performances on the data collected by the Beijing Traffic Management Bureau. Yang et al. [46] combine a Bi-LSTM network with a Conditional Random Field (CRF) model for Chinese speech recognition. The above studies give a fruitful insight into how LSTMs are effective models, and into how there are many practical applications related to the problem of time series analysis.

## 3. Basic Concepts

### 3.1. k-Means Clustering Algorithm

**Figure 1:**
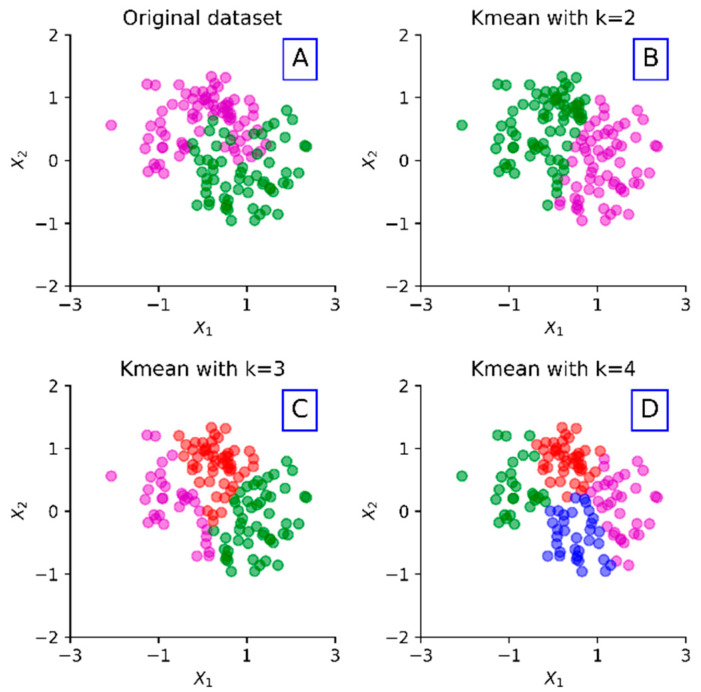
Results of the *k*-means clustering algorithm with various *k* values on an example dataset.

Unsupervised learning is one category of machine learning tasks, which is used to draw inferences from unlabeled datasets. Typically, unsupervised learning employs a clustering technique to group unlabeled observations based on one of several similarity measures such as *Euclidean*, *Cosine*, *Jaccard*, and *Manhattan* distances. The most popular unsupervised learning algorithm is the *k*-means clustering algorithm. The *k*-means algorithm has been successfully used in customer segmentation in economics [48,49], computer vision [50], and many other domains. Basically, the *k*-means algorithm aims to assign *n* observations in the dataset into *k* (≤ *n*) disjoint sets, **S** = {$S_{1}$, $S_{2}$, …, $S_{k}$}, by solving the optimization problem as follows:

(1) $$J=\overset{m}{\underset{i=1}{\sum }}\overset{K}{\underset{k=1}{\sum }}w_{ik}\|x^{i}-\mu_{k}{\|}^{2},$$

where $w_{ik}$ = 1 for the observation $x^{i}$ belonging to the cluster $S_{k}$; otherwise, $w_{ik}$ = 0. Meanwhile, $\mu_{k}$ is the centroid of the cluster $S_{k}$, which consists of the observation $x^{i}$. Figure 1 shows an example of the *k*-means clustering algorithm that utilizes *Euclidean* distance on an example dataset with various *k* values.

### 3.2. Long Short-Term Memory Networks

**Figure 2:**
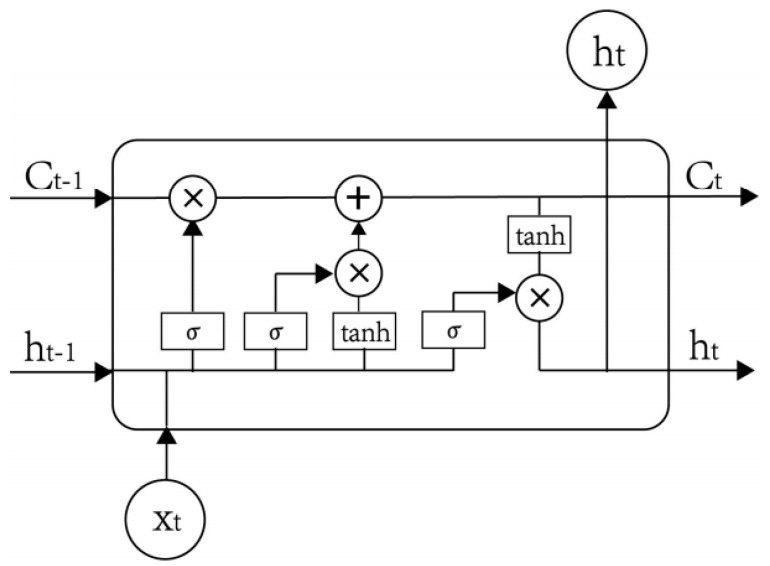
The diagram of an LSTM memory cell at the time step *t*.

The Long Short-Term Memory (LSTM) network, a special kind of RNN, is capable of learning long-term dependencies. LSTM was first introduced by Hochreiter and Schmidhuber [51]. An LSTM model utilizes a unique set of memory cells instead of the hidden layer neurons in traditional RNN models. LSTM filters information through the gate structure to maintain and update the state of memory cells. There are three types of gate structures, including input, forget, and output gates. Each memory cell employs two types of nonlinear activation functions, including a *sigmoid* (σ(·)) function and a *tanh* function. Figure 2 presents the diagram for an LSTM memory cell at the time step *t*.

First, the forget gate in an LSTM memory cell identifies which cell state information will be discarded. As shown in Figure 2, the memory cell takes the output of the previous step $h_{t-1}$ and the external information at the current step $x_{t}$ as inputs. Then, this gate combines them into a long vector through the *sigmoid* function as follows:

(2) $$f_{t}=\sigma \left(W_{f}\cdot \left[h_{t-1},\;x_{t}\right]+b_{f}\right).$$

In Equation (2), $W_{f}$ and $b_{f}$ are the weight matrix and bias vector of the forget gate, respectively. The forget gate’s main function is to record how much the cell state $C_{t-1}\;$of the previous step is reserved to the cell state $C_{t}$ of the current step. The output of this gate is a value ranging from 0 to 1, where 1 indicates the complete reservation while 0 indicates the complete discernment.

Furthermore, the input gate decides how much of the current moment input $x_{t}$ is reserved into the cell state $C_{t}$. This gate prevents useless information entering the memory cells. This gate consists of two functions, as follows. Equation (3) aims to find the state of the cell that must be updated, which is determined by the *sigmoid* function. Equation (4) serves to update the information to the cell state. In this function, a new candidate vector $C_{t}^{’}$ is created through the *tanh* function to control how much new information will be added. Equation (5) is utilized to update the cell state of the memory cells.

(3) $$i_{t}=\sigma \left(W_{t}\cdot \left[h_{t-1},\;x_{t}\right]+b_{t}\right),$$

(4) $$C_{t}^{’}=\tan h\left(W_{c}\cdot \left[h_{t-1},\;x_{t}\right]+b_{c}\right),$$

(5) $$C_{t}=f_{t}\cdot C_{t-1}+i_{t}\cdot C_{t}^{’}.$$

Finally, the output gate controls how much of the current cell state is discarded. The output information,$\;o_{t}$, is first identified by the *sigmoid* function:

(6) $$o_{t}=\sigma \left(W_{o}\cdot \left[h_{t-1},\;x_{t}\right]+b_{o}\right).$$

Then the cell state is processed by *tanh* and multiplied by the output information $o_{t}$ to obtain the final output portion, which is formulated as:

(7) $$h_{t}=o_{t}*\tan h\left(C_{t}\right).$$

### 3.3. Transfer Learning

Training neural networks have faced two critical problems, including expensive resources and computational costs. Because training a neural network requires numerous matrix operations and expensive resources, the resource costs would be extremely high if we performed a similar process again for different models. Besides, the computational time to train a number of deep learning models increases exponentially when the deep neural networks become deeper and more complex. The idea of transfer learning [52,53,54] is introduced to overcome the problems of expensive resources and computational costs for training multiple deep learning models. Transfer learning methodology focuses on applying the gained knowledge of deep learning models from a trained architecture to train another deep learning model on a different task. Specifically, this methodology first trains a base network on a source dataset, and then it transfers the weights of the base network to a target network. In conclusion, instead of training the new neural network model from scratch, this methodology “transfers” the learned knowledge from a base network model. In our study, we assume that each time series shares characteristics such as trends and periodicities with the remaining one. For example, every Saturday and Sunday the energy consumption of all smart buildings is decreased; hence, the decreasing trend and the repeated demand patterns on the weekend are shared between all building apartments. Therefore, it is intuitive to apply the transfer learning mechanism to our problem.

**Figure 3:**
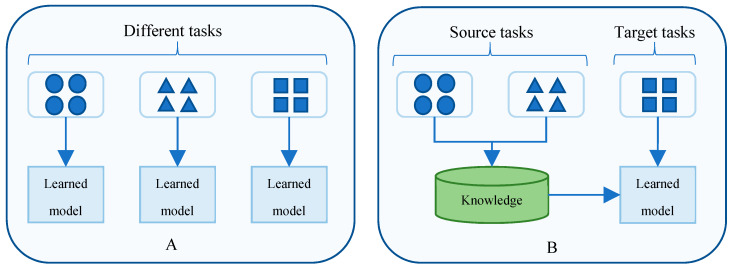
The comparison between (**A**) traditional machine learning and (**B**) transfer learning.

Figure 3 shows the comparison between traditional machine learning and transfer learning. Traditional machine learning algorithms (Figure 3A) learn from an individual dataset, and each traditional machine learning model works independently. Meanwhile, transfer learning (Figure 3B) utilizes the knowledge gained from multiple source domains’ datasets to transfer to the target domain. Hence, transfer learning reduces the computational time, while also improving the predictability performances. 

In the problem of multiple electric energy consumption forecasting in smart buildings raised in this article, training multiple LSTM models is dramatically time and system resource consuming. Therefore, this study employs the concept of transfer learning and combines transfer learning with a cluster-based strategy for training LSTM models to overcome the time-consumption problem.

## 4. Materials and Methods

### 4.1. The Experimental Datasets

**Figure 4:**
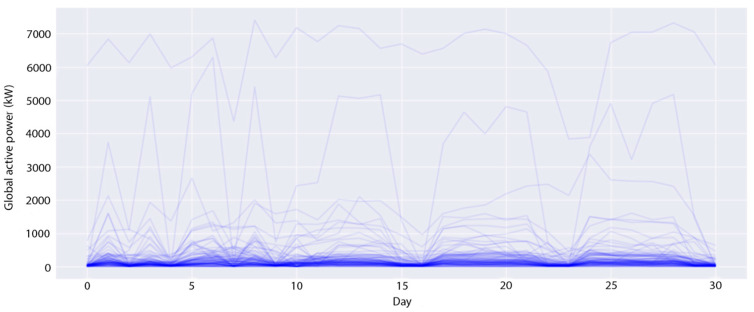
All the daily-load profiles in 30 days of the *B1* dataset.

**Figure 5:**
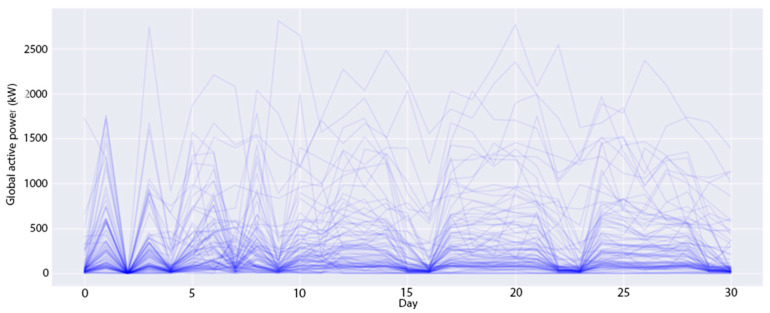
All the daily-load profiles in 30 days of the *B2* dataset.

The experimental datasets are collected from two smart buildings located in South Korea. These datasets contain 15-min demand loads of a number of apartments (profiles) in these buildings over three years, from January 2016 to December 2018. The first building consists of 96 profiles (*B1* dataset), whereas the second building has 91 profiles (*B2* dataset). The demand of a profile is the amount of energy used by all electronic devices such as computers, office equipment, lighting and air conditioning, etc. at a specific time; it is measured in kilowatts (kW). In the transformation module, this study converts these datasets to daily demand loads by summing all 15-min demand loads of all profiles on the same date. Figure 4 and Figure 5 show the daily-load profiles in the first 30 days of the *B1* dataset and of the *B2* dataset, respectively. In the *B1* dataset, there are several profiles with a huge daily power consumption of over 5000 kW, while most other profiles are around 100 kW per day. Meanwhile, the profiles in the *B1* dataset are evenly distributed from 0 to 1000 kW per day. From the above analysis, the optimal number of clusters is different for each experimental dataset. Therefore, we perform the first experiment to identify the best number of clusters. Note that this study utilizes the time series in the first two years for training and in the last year for testing. This experiment only considers the training datasets to find the optimal number of clusters.

### 4.2. The MEC-TLL Framework

**Figure 6:**
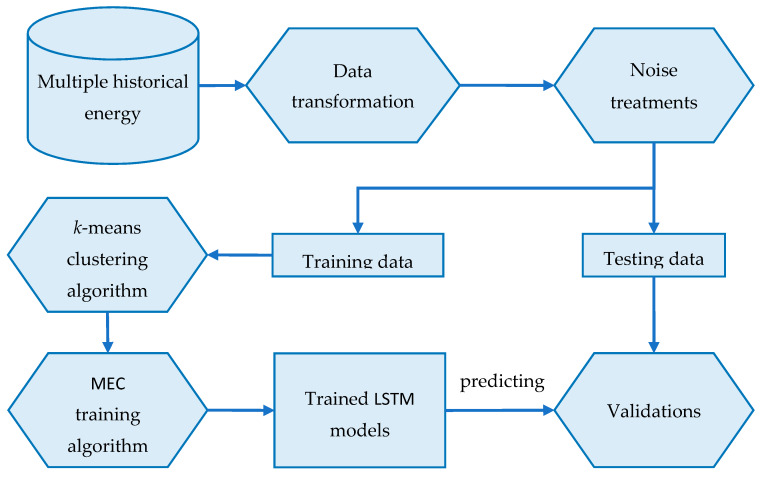
The overall architecture of the MEC-TLL framework.

This section introduces an effective framework for Multiple Electric Energy Consumption forecasting in a smart building using Transfer Learning and LSTM, denoted by MEC-TLL. The overall architecture of the proposed framework is shown in Figure 6. In the preprocessing phase, MEC-TLL first converts the data from multiple historical energy consumption datasets to numbers of time series in the daily demand load that indicate the daily energy consumption of all profiles in the smart building. Next, we apply several noise treatment techniques to remove noise or incomplete data. 

To evaluate our proposed framework, this study then divides the time series energy consumption datasets into training and testing data. In the next phase, we use the *k*-means clustering algorithm to cluster the training data into several clusters. To determine the number of clusters, we perform a *Silhouette* analysis [55,56], which is to interpret and to validate the consistency within clusters of data. *Silhouette* is a measure of how similar a point is to its own cluster compared to other clusters. This method provides a graph containing several scores, which range from −1 to 1. A high score indicates that a point has a good match with the cluster it belongs to. The detailed analysis of this experiment for each dataset is presented in Section 4.2. After this step, the training data is divided into disjoint clusters, which will be passed to the Multiple Electric Energy Consumption prediction (MEC) training algorithm. The pseudocode of this algorithm is presented in Algorithm 1.

The computation of the MEC training algorithm is summarized as follows. The input of this algorithm is *n* clusters, which are processed in the previous step. For each cluster in the training dataset, the algorithm will train an LSTM model using the center point time series, which is denoted as *LSTM_Model_base_* (Lines 2–4). Then, the algorithm uses *LSTM_Model_base_* as a base model for training the remaining profiles by using transfer learning to reduce the computation time (Line 5). Finally, the algorithm returns the LSTM models of all profiles (Line 6). Due to the advantages of transfer learning, our proposed framework reduces the overall computation time. Hence, our method reasonably outperforms traditional machine learnings w.r.t. computational time.

Finally, the trained LSTM models are used to predict the testing data. The validation module compares the predicted values and the actual values to validate the proposed framework by several performance metrics as well as the computational time.
**Algorithm 1.** MEC training algorithm**Input:**
*n* clusters **Output:**
*n* trained LSTM models
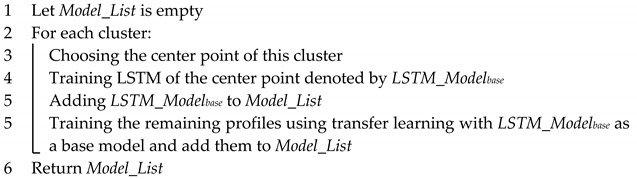


## 5. Results

### 5.1. Experimental Setting

LSTM models are implemented with Keras library, an open-source neural-network library in Python. In addition, the *k*-means clustering algorithm with the *Silhouette* analysis is provided by Scikit-learn package [57], an open-source machine learning library. All experimental methods are performed on a server containing four GTX 1080 Ti. LSTM models in all experimental approaches are trained in 30 epochs, and a batch size at 30 using Adam optimization [58], an adaptive learning rate optimization algorithm, with the initial learning rate as 0.005. Meanwhile, the transferred LSTM models are also trained in 30 epochs, and a batch size of 30 using Adam optimization with the initial learning rate as 0.001. It is better to choose a small learning rate for transfer learning because high learning rates increase the risk of losing previous knowledge. Therefore, we use the learning rate at 0.001 for transfer learning and 0.005 for traditional LSTM models.

To compare the predictive performance, this study uses three common metrics, namely *RMSE*, *MAE*, and *MAPE*, which are usually used to evaluate time series forecasting models. The first metric, *RMSE*, is the standard deviation of prediction errors. Let the residuals be a measure of how far from the regression line the data samples are. *RMSE* is a measure of how spread out these residuals are, which is formulated as:

(8) $$RMSE=\sqrt{\frac{1}{n}\;\overset{n}{\underset{1}{\sum }}{\left(y-\hat{y}\right)}^{2}.}$$

The second metric, *MAE*, measures the average magnitude of the prediction errors without considering their directions. This metric uses the same weight for all prediction errors as follows:

(9) $$MAE=\frac{1}{n}\;\overset{n}{\underset{1}{\sum }}\left|y-\hat{y}\right|.$$

The third metric, *MAPE*, is a measure of the prediction accuracy in percentage of the following equation:

(10) $$MAPE=\frac{100\%}{n}\;\overset{n}{\underset{1}{\sum }}\left|\frac{y-\hat{y}}{y}\right|.$$

This section conducts the comparisons of the above metrics and the computational time on the two experimental datasets between MEC-TLL and the two following approaches to show the effectiveness of the proposed approach. The first approach, namely TML-LSTM, is a traditional machine learning and employs LSTM models to predict the electric energy consumption for each profile. Therefore, the TML-LSTM approach has to train 96 LSTM models in the *B1* dataset and 91 LSTM models in the *B2* dataset. The second approach, TL-LSTM, employs transfer learning without any cluster-based algorithm for clustering profiles. In this approach, we randomly choose a profile and train the base model using this profile. Then, we use the base model as the pre-trained model for the transfer learning module. TL-LSTM is created to show the effectiveness of the cluster-based strategy for transfer learning that is applied in the MEC-TLL approach.

### 5.2. Silhouette Analysis

**Figure 7:**
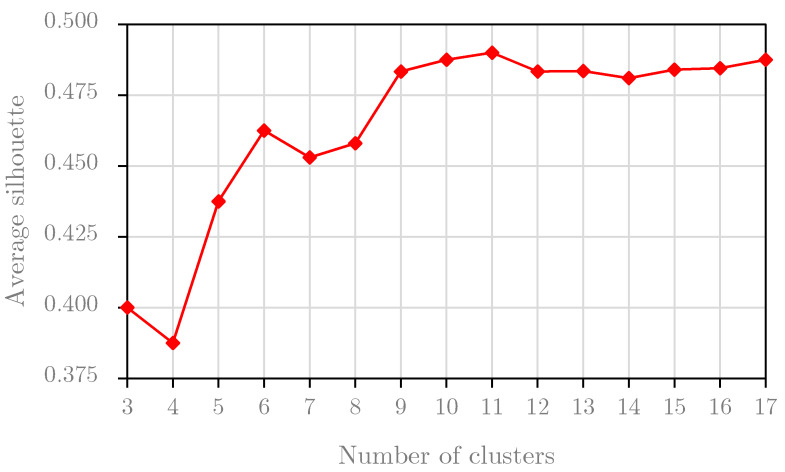
The average silhouette score per number of clusters for the *B1* dataset.

**Figure 8:**
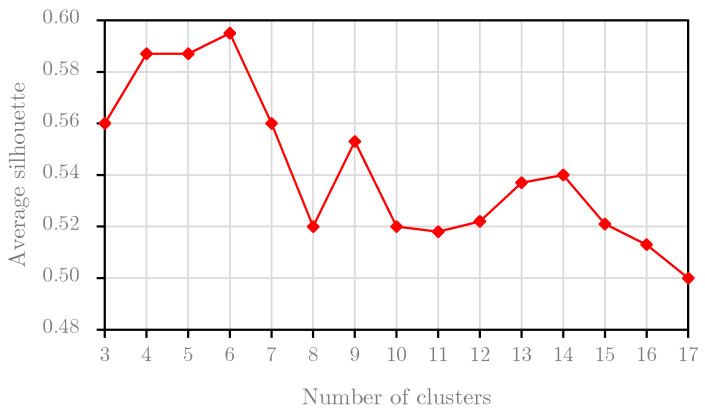
The average silhouette score per number of clusters for the *B2* dataset.

This section performs the *Silhouette* analysis for two experimental datasets. *Silhouette* analysis serves to find the separation distances between resulting clusters. The result of this analysis, the *Silhouette* plot, shows a measure of the closeness between each sample in one cluster and samples in the neighboring clusters. Based on this plot, we can identify the best number of clusters. We perform the *Silhouette* analysis for the first two years of the *B1* dataset, i.e., the training the *B1* dataset, and we obtain the graph result in Figure 7. Based on the results, we choose the optimal number of clusters for the *B1* dataset at 11 because the average *Silhouette* score reached the highest value with the cluster number of 11. For the *B2* dataset, the result of the *Silhouette* analysis for the training set of the *B2* dataset is presented in Figure 8. Obviously, we set the optimal number of clusters for the *B2* dataset to 6 because the graph in Figure 8 peaks at this value.

In conclusion, this experiment indicates that the optimal numbers of clusters are different values with different datasets. For this study, this experiment selected the optimal numbers of clusters at 11 and 6 from the training sets for the *B1* dataset and *B2* dataset, respectively. These values are also used in the second experiment.

### 5.3. Experimental Results and Discussions

The second experiment is conducted to compare the averages of several performance metrics, including *RMSE*, *MAE*, *MAPE*, and the computational time among the experimental methods. 

**Figure 9:**
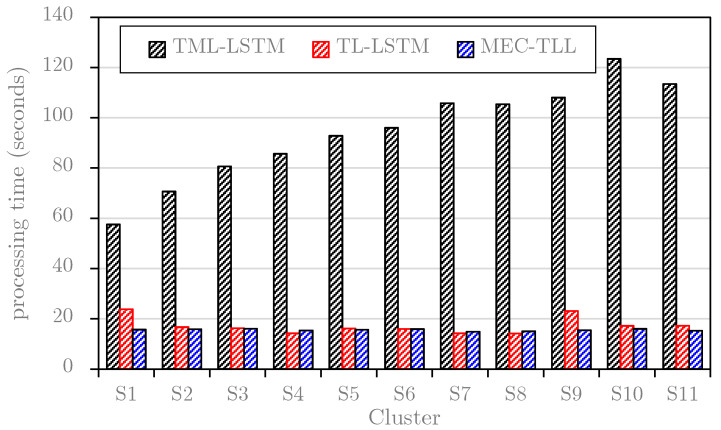
The computational time of each cluster on the *B1* dataset.

**Figure 10:**
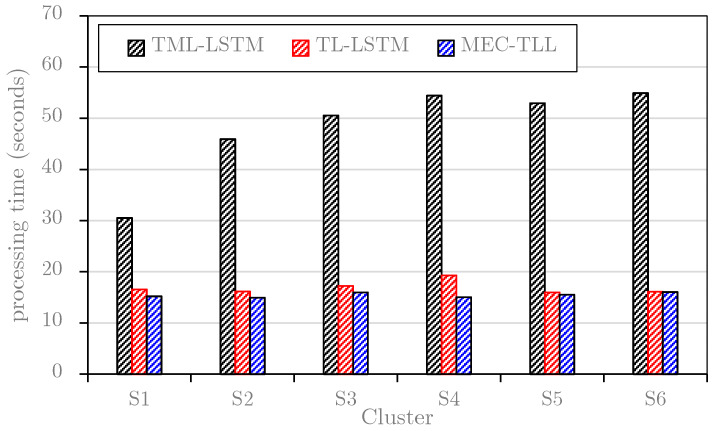
The computational time of each cluster on the *B2* dataset.

This study first plots the average learning and predicting times of TML-LSTM, TL-LSTM, and MEC-TLL approaches for each cluster on the *B1* dataset (see Figure 9) and *B2* dataset (see Figure 10). Note that these graphs only consider the training and predicting time for each cluster in the *B1* dataset and *B2* dataset. In general, the results in both figures are consistent, where TL-LSTM and MEC-TLL obviously outperform TML-LSTM. In particular, Figure 9 shows that the average computational times of TML-LSTM range from 57 s to 123 s, whereas the average computational times of TL-LSTM and MEC-TLL range from 15 s to 23 s. Therefore, the transfer learning-based approaches improve at least 75% of the computational time compared to the traditional machine learning approach on the *B1* dataset. In Figure 10, the average computational times of TML-LSTM range from 30 s to 54 s, whereas the average computational times of TL-LSTM and MEC-TLL range from 15 s to 19 s. Therefore, the transfer learning-based approaches improve at least 50% of the computational time compared to the traditional machine learning approach on the *B2* dataset. 

Table 1 and Table 2 show the averages of *RMSE*, *MAE*, *MAPE*, and the computational time of the experimental methods on the *B1* dataset and *B2* dataset, respectively. To obtain the average computational time of MEC-TLL, this study first computes the computational time that includes the clustering time with the optimal number of clusters (11 for the *B1* dataset and 6 for the *B2* dataset), the learning time, and the prediction time for the whole dataset. Then, the computational time of MEC-TLL will be divided by the number of profiles (96 for the *B1* dataset and 91 for the *B2* dataset) to obtain the averages. In other words, we include the cluster time with the optimal number of clusters (7.2 s for the *B1* dataset, and 2.9 s for the *B2* dataset) in the overall computational time of MEC-TLL, and then take the average.

For the *B1* dataset, the proposed approach, MEC-TLL, is the best approach in terms of predictive performances with 1.142, 0.670, and 34.32 for *RMSE*, *MAE*, and *MAPE*, respectively. In addition, MEC-TLL also achieves impressive results on the average computational time on the *B1* dataset compared with the TML-LSTM approach. For details, the TML-LSTM approach is extremely time-consuming with 101.4 s for each profile in the *B1* dataset. Therefore, TML-LSTM takes 2.7 h on the entire *B1* dataset. Meanwhile, the proposed method and TL-LSTM take around 25 min for the whole *B1* dataset. 

In Table 2, the achieved results for the *B2* dataset are similar to the *B1* dataset. The MEC-TLL framework obtains a 60% reduction in the average computational time compared with the TML-LSTM approach. Meanwhile, the average computational times of MEC-TLL and TL-LSTM are almost equivalent to 15.4 s and 16.8 s, respectively. In addition, the predictive performances of TML-LSTM and MEC-TLL are almost the same in terms of *RMSE*, *MAE*, and *MAPE*. Meanwhile, TL-LSTM has a poor predictability compared to TML-LSTM and MEC-TLL.

In summary, the experimental results indicate that the transfer learning-based approaches, i.e., TL-LSTM and MEC-TLL, show a great improvement in computational time compared with traditional machine learning approaches, i.e., TML-LSTM. The cluster-based strategy helps the MEC-TLL approach in achieving better predictive performances than the TL-LSTM approach. In addition, the predictive performances of TML-LSTM and MEC-TLL, including *RMSE*, *MAE*, and *MAPE*, are almost the same, while the predictive performances of TL-LSTM decrease insignificantly. Therefore, our method (MEC-TLL) is the best of the empirical methods in terms of computational time, limited resources, and predictability.

## 6. Conclusions

This study develops an effective framework for multiple electric energy consumption forecasting in smart buildings, namely MEC-TLL, which utilizes the concept of transfer learning and a cluster-based strategy for training the LSTM models to reduce the computational time. This framework first clusters the time series training set to several clusters using the *k*-means clustering algorithm. For each cluster obtained from the previous phase, we train the LSTM model using the centroid and use the trained model as the base model for transfer learning to the remaining profiles. To verify the effectiveness of our framework, we conduct two experiments on two real-world datasets collected from two smart buildings in South Korea. The first experiment, based on a *Silhouette* analysis, is to identify the optimal number of clusters for each experiment dataset. This experiment found that the optimal number of clusters is 11 and 6 for the *B1* dataset and *B2* dataset, respectively. Then, we utilize the optimal number of clusters for the MEC training algorithm in the second experiment. The results of the second experiment confirm that our approach outperforms the traditional machine learning approach and an approach employing transfer learning without the cluster-based strategy for multiple electric energy consumption forecasting in terms of the computational time, while also retaining a predictive performance.

In the future, we will continue to enhance the performance of multiple electric energy consumption forecasting in terms of computational time as well as the predictive performance by using several modern techniques such as Bi-directional Long Short-Term Memory for time series forecasting, Discrete Wavelet Transform (DWT), and Discrete Fourier Transform (DFT) for time series feature extraction. In addition, we will try to adapt the proposed framework to real-time environments for industrial applications.

## Figures and Tables

**Table 1:** Performance results of the experimental methods on the *B1* dataset.

Approaches	The Average Computational Time (Seconds)	RMSE	MAE	MAPE
TML-LSTM	95.4	1.172	0.721	35.24
TL-LSTM	17.2	1.564	0.821	40.34
MEC-TLL	15.7	1.142	0.670	34.32

**Table 2:** Performance results of the experimental methods on the *B2* dataset.

Approaches	The Average Computational Time (Seconds)	RMSE	MAE	MAPE
TML-LSTM	48.2	1.425	0.912	41.42
TL-LSTM	16.8	1.718	1.054	45.21
MEC-TLL	15.4	1.368	0.891	41.07
